# *Haemonchus contortus* infection alters parasitological, hematological, and colostrum profiles with metabolomic and lipidomic signatures in Florida cracker ewes during the peripartum period

**DOI:** 10.3389/fvets.2026.1770252

**Published:** 2026-04-21

**Authors:** Sola J. Ikuejamoye-Omotore, Makenzie Harrison, John O. Adebayo, Anijia Mills-Widemon, Uchenna Y. Anele, Andrea Gentry-Apple, Zaira M. Estrada-Reyes, Scott Bowdridge, Ibukun M. Ogunade, Andres A. Pech-Cervantes, Thomas H. Terrill, Alexis Ruiz-González

**Affiliations:** 1 Department of Animal Science, North Carolina Agricultural and Technical State University, Greensboro, NC, United States; 2 College of Agriculture, Food and Natural Resources, CAFNR Research, Prairie View A&M University, Prairie View, TX, United States; 3 Division of Animal and Nutritional Science, West Virginia University, Morgantown, WV, United States; 4 College of Agricultural, Family Sciences, and Technology, Fort Valley State University, Fort Valley, GA, United States; 5 Department of Animal Science, Laval University, Québec City, QC, Canada

**Keywords:** colostrum, Florida Cracker ewes, Haemonchus contortus, hematology, lipidomics, parasitology, metabolomics

## Abstract

This study evaluated for the first time the effects of *H. contortus* infection on hematological and parasitological parameters, as well as on the composition, metabolomic, and lipidomic profiles of colostrum in periparturient Florida Cracker ewes. Twenty pregnant Florida Cracker ewes were allocated to infected (INF, *n* = 10) or control (CTL, *n* = 10) groups at 90 days of pregnancy. Then, at 120 days of gestation, the INF group received an oral dose of 10,000 L3 *H. contortus* larvae, while the CTL group received 3 mL of distilled water. Fecal egg counts (FEC) and FAMACHA scoring were performed before infection (−1 h), and at 7-, 14-, 21-, 28- and 35- days post-infection (pi). Blood was collected by jugular venipuncture before infection (−1 h) and at 3-, and 6-h, and at 7-, 14-, 21- and 28-days pi from all ewes for full hematology analysis. Colostrum samples were collected at lambing from each ewe. The FEC data was log transformed [log_10_(FEC + 100)] for analysis (LFEC). A mixed-effects model with repeated measures was used to analyze the hematological and parasitological data. Colostrum chemical composition was analyzed using a *t*-test, while metabolomic and lipidomic data were processed using MetaboAnalyst 5.0. Parasitological analysis revealed significant differences for LFEC and FAMCHA score, at 21-, 28- and 35-days pi and at 14–21-, 28- and 35-days pi, respectively. For hematological data, significant differences (*p* ≤ 0.05) were observed for white blood cell count (WBC), lymphocyte count (LYM), neutrophil count (NEU), monocyte count (MON) and mean corpuscular volume (MCV). No significant differences were observed for the chemical composition of colostrum. For colostrum metabolomics, 23 differentially abundant metabolites (*p* ≤ 0.05, FC ≥ 1.5 or ≤ 0.67) were observed between the experimental groups. Lipidome analysis identified 4,702 lipid species in the colostrum samples. Biomarker analysis identified 3 lipid species (FA 15:1; O, MG 20:5, PE 41:6) as potential biomarkers between the INF and CTL groups. These lipids support cellular integrity and energy biogenesis. These findings highlight the impact of *H. contortus* infections on colostrum composition in Florida Cracker ewes, suggesting the need for further research to understand how these changes impact the metabolism and performance of lambs.

## 1 Introduction

*Haemonchus contortus* is one of the greatest health concerns of small ruminants (1). This blood-sucking parasite has a more profound impact on pregnant ewes, who become particularly vulnerable to haemonchosis during the peripartum period (late pregnancy and early lactation) due to the suppression of immunity during this stage (2). Infection with *H. contortus* has been demonstrated to significantly alter the biochemical, hematological, and plasma metabolic status of the host (3, 4). The altered maternal metabolic state influences the circulating plasma constituents required for colostrum synthesis (5) as colostrum constituents are translocated from blood plasma to the mammary epithelial cells during synthesis (5, 6). Colostrum supplies lambs with essential nutrients and immunity, and inadequate quantity and quality can impede the transmission of passive immunity transfer (FPT) leading to increased morbidity and mortality (7, 8). Colostrum constituents include fat, lactose, proteins, immunoglobulins, vitamins, minerals, hormones, growth factors, cytokines, and enzymes (9). A major component of colostrum are fatty acids which newborn lambs require for heat generation (thermoregulation) and supply of many bioactive compounds (6, 10).

It has been demonstrated that gastrointestinal nematodes reduce the overall amount of milk produced by infected sheep (11–13). However, the effect of *H. contortus* infections on colostrum composition is still not well defined (11, 14), particularly in colostrum from parasite resistant breeds such as Florida Cracker sheep. Omics techniques such as metabolomics and lipidomics are gaining relevance in livestock research allowing scientists to quantify thousands of low-abundant metabolites and lipid molecules present in biological samples (15–17). These techniques provide insights into biological alterations under different stressors (18–21). Therefore, the objective of this study was to determine the effect of *H. contortus* infection on the chemical composition and the metabolomic, and lipidomic profile of colostrum in Florida Cracker ewes.

## 2 Materials and methods

### 2.1 Animal population and treatment allocation

All protocols used in this experiment were approved by the Institute of Animal Control and Use Care (LA22-0003) at North Carolina Agricultural and Technical State University. A total of 20 Florida Cracker ewes (two-year-old) were used. Ewes were synchronized for estrus using a commercial progesterone (0.3 g P4) intravaginal insert (EAZI-BREED CIDR Sheep Insert, Zoetis Animal Health, Florham Park, NJ) and randomly assigned to two covered concrete floor pens. The intravaginal inserts were removed after 10 days, and two rams of the same breed were immediately introduced into each ewe pen. Each ram was fitted with a marking harness and was single-sire mated to ewes within the same pen group, using an ewe-to-ram ratio of 10:1. Ewes were exposed to rams for a 30-day breeding season. A pregnancy blood test was conducted on day 30 after ram introduction to confirm pregnancy. For this test, blood samples were collected by jugular venipuncture and using EDTA vacutainer tubes and 21 g needles. Blood samples were sent to a commercial veterinary lab (Cool Springs Mobile Vet Service, Cleveland, NC, USA) for analysis and confirmation of pregnancy. All ewes were confirmed pregnant after analysis. Deworming of the ewes was carried out using a combination of Cydectin (0.2 mg/kg of body weight) and levamisole (8 mg/kg of body weight) at 90 days of gestation. Ten days following treatment, a fecal egg count reduction test was conducted. Then, ewes were randomly reassigned to infected (INF, *n* = 10) or control (CTL, *n* = 10) groups and were housed in two new covered concrete floor pens, with similar dimensions and characteristics, respectively. Animals were fed a combination of commercial sheep pellet (20% protein) and alfalfa hay with *ad libitum* water during the whole pregnancy. The INF group received an oral dose of 10,000 L3 *H. contortus* larvae, while the CTL group received 3 mL of distilled water at 120 days of gestation, respectively.

### 2.2 Collection of parasitological and hematological parameters

**Figure 1:**
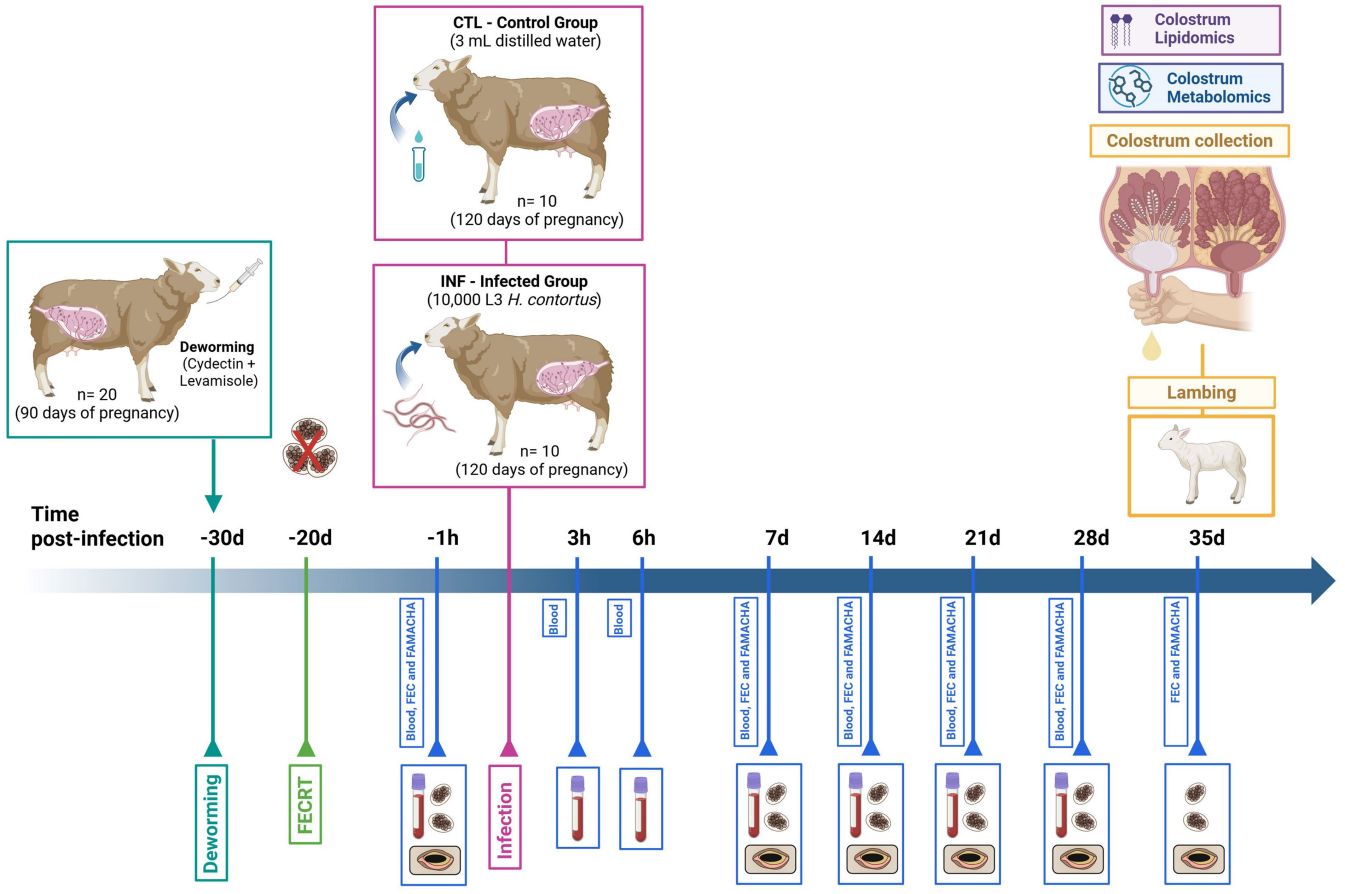
Representation of the artificial infection trial with *Haemonchus contortus* L3 larvae in pregnant Florida Cracker ewes. Pregnant Florida Cracker ewes were initially dewormed, followed by a fecal egg count reduction test conducted 10 days post-treatment. Measurements of fecal egg count (FEC) and FAMACHA scores were recorded 1 hour prior to infection (−1 h), and subsequently at 7, 14, 21, 28, and 35 days post-infection (pi). Blood samples were collected at −1, 3, 6 h, and at 7, 14, 21, and 28 days pi for comprehensive hematological analysis. At lambing, colostrum samples from both experimental groups were obtained for metabolomic and lipidomic profiling. Image was created with BioRender. Estrada, Z. (2026) https://BioRender.com/tx0p6h3.

Collection of parasitological measures is represented in Figure 1. Fecal egg count (FEC) and FAMACHA score were performed before infection (−1 h), and at 7-, 14-, 21-, 28-, and 35-days post-infection (pi). For FEC, approximately 2 g of fecal samples were collected directly from the rectum of each experimental ewe using antiseptic lube and plastic bags and transported to the laboratory for analysis. The fecal samples were analyzed using the Mc Master technique as described by Paras et al. (22). For FAMACHA, the color of ewe’s eyelid conjunctiva was compared to the FAMACHA card (23). Blood was collected by jugular venipuncture using vacutainer EDTA tubes and 21-gauge needles before infection (−1 h) and at, 3-, and 6-h, and at 7-, 14-, 21- and 28-days pi from all ewes. A full hematology analysis was performed using the Vetscan HM5 system (Zoetis, Parsippany, New Jersey).

### 2.3 Colostrum collection and composition analysis

Collection of colostrum is represented in Figure 1. Colostrum samples (55 mL per ewe) from all 20 ewes were collected immediately after lambing using a milk collector (Udderly EZ, Uderly Animal Products, KY, USA). From these collected samples, a total of 50 mL of colostrum per ewe was used for composition analysis, and 5 mL was used for metabolomics and lipidomics analyses, respectively. For composition analysis, a milk analyzer (Lactoscope 300, Perkin Elmer, Waltham, MA, USA) was used to evaluate the overall content of fat, protein, lactose, and total solids.

### 2.4 Colostrum metabolomics

Methanol extraction was performed on colostrum samples collected from all ewes as described by Zhao et al. (24). The extract was stored at −80°C until needed for metabolite analysis. The colostrum samples underwent untargeted metabolomic analysis using chemical isotope labeling (CIL) and are coupled with liquid chromatography-mass spectrometry (LC–MS) for high-performance metabolome analysis. This technique employs differential ^12^C-/^13^C-isotope labeling to derivatize metabolites according to their chemical groups (such as amines/phenols, carboxylic acids, hydroxyls, and carbonyls). Comprehensive details of the methods, including sample preparation and analysis, have been reported by Zhao et al. (24). Quality control (QC) data supporting metabolite detection and data preprocessing steps are provided in Supplementary material S1. The raw LC–MS data files were processed using IsoMS Pro 1.2.16 to eliminate duplicate peaks associated with the same metabolite, including dimers, multimers, and adduct ions. Equal volume mixture of a (^12^C-labeled and a ^13^C-labeled pooled samples) was used as quality control. The raw LC–MS data files were processed using IsoMS Pro 1.2.16 to eliminate duplicate peaks associated with the same metabolite, including dimers, multimers, and adduct ions. Metabolite identification was performed using a two-tiered identification strategy. In tier 1, peak pairs derived from metabolite intensity tables were matched against a chemical isotope labeling (CIL) metabolite library based on accurate mass and retention time (RT) information (25). This library currently comprises 1,060 unique endogenous metabolites, including 711 amines/phenols, 187 carboxyls, 85 hydroxyls, and 77 carbonyls. In tier 2, a linked identity library was used to annotate the remaining peak pairs based on accurate mass and predicted RT. This linked identity library contains over 2,000 metabolites associated with metabolic pathways and was curated from the Kyoto Encyclopedia of Genes and Genomes (KEGG) database (26). Peak pairs that could not be annotated at either tier were classified as unidentified metabolites. Metabolites for the peak pairings were identified by comparing mass and retention time with the linked identity libraries and CIL, as described by Huan and Li (25) and Idowu et al. (27).

### 2.5 Colostrum Lipidomics

Lipids were extracted using a modified Folch liquid–liquid extraction procedure that included methanol and dichloromethane. Colostrum aliquots from the INF (*n* = 10) and CTL (*n* = 10) groups were pooled to serve as quality control. A detailed description of these protocols has previously been reported by (28). Pretreatment and treatment steps were done as described by Oyebade et al. (29). An ultra-high-performance liquid chromatograph (Thermo Fisher Scientific) coupled to a Bruker Impact II quadrupole time-of-flight mass spectrometer (Bruker Daltonics) for the negative and positive ions was used to perform lipidome analysis of the extracted colostrum samples. The sample LC–MS data and its quality controls were processed in positive and negative ionization to generate 1 feature intensity table. Quality control (QC) data supporting lipid detection and data preprocessing steps are provided in Supplementary material S2. Lipids were identified using a three-tiered identification process that included precise mass matching and MS/MS identification (28). For tier 1 and tier 2 annotations, putative metabolite identification was conducted using tandem mass spectrometry (MS/MS) by matching spectra against the MS-DIAL LipidBlast library,^1^ the Human Metabolome Database (HMDB; https://hmdb.ca), and the MassBank of North America (MoNA) LC–MS/MS libraries,^2^ all integrated within MetaboScape software (version 4.0). Features that could not be annotated at tiers 1 or 2 were subsequently queried against the LipidMaps database^3^ for putative identification based on accurate mass matching (tier 3). Identification criteria included an MS/MS match score ≥ 500 and precursor m/z error ≤ 5.0 mDa for tier 1, an MS/MS match score ≥ 100 and precursor m/z error ≤ 5.0 mDa for tier 2, and accurate mass matching with an m/z error ≤ 5.0 mDa (≤ 20 ppm) for tier 3. The identification was further optimized using a 6-tier filtering and scoring approach as detailed in Drotleff et al. (30). Data normalization was done using Lipdomix (Splash Lipidomix Mass Spec Standard, Avanti Polar Lipids), a quantitative combination of deuterated lipids from several lipid classes (28).

### 2.6 Statistical analysis

The Shapiro–Wilk test (31) was used to check for normality of the parasitological, hematological and colostrum chemical composition data. The FEC data was log transformed [log_10_ (FEC + 100)] for analysis (LFEC). The value of 100 was added to account for zeros in the analyses to facilitate statistical processing. A mixed model with repeated measures was used for parasitological and hematological data. Fixed effects included treatment (trt) group (INF vs. CTL), time (time post-infection), and the treatment × time interaction (trt x time). Animal was modeled as a random effect, and baseline measurements were incorporated as a covariate to adjust for initial variability (32). The unstructured variance–covariance structure provided the best model fit, as indicated by the lowest values for the Akaike Information Criterion (AIC = 598.1), and the corrected Akaike Information Criterion (AICc = 3.4). For the colostrum chemical composition data, a t-test was used to analyze it in Rstudio. Statistical analysis of the metabolome and lipidome colostrum data was performed with Metaboanalyst 5.0 software (33). The metabolome and lipidome data were normalized by median, square root transformation, and auto scaling. Principal Component Analysis (PCA) was conducted to visualize the difference between the treatment groups, respectively (34). A volcano plot analysis (univariate analysis) was performed to classify differentially abundant metabolites and differentially abundant lipid species (*p* ≤ 0.05, FC ≥ 1.5 or ≤ 0.67) in colostrum samples. We recognize that no universally accepted FC threshold exists for metabolomics or lipidomics analyses and that threshold selection involves a trade-off between sensitivity and specificity. While more stringent FC cutoffs may reduce false positives, they may also exclude moderate but potentially relevant metabolic changes in tightly regulated animal systems. For biomarker analysis, a receiver operating characteristic (ROC) curve calculated by the ROCCET web server was used to identify differentially abundant biomarkers that contributed most to the difference (AUC > 0.80 and *p* ≤ 0.05) between the treatment groups. Pathway enrichment analysis was performed to elucidate the enriched metabolic pathways between the treatment groups.

## 3 Results

### 3.1 Parasitological and hematological measures

**Figure 2:**
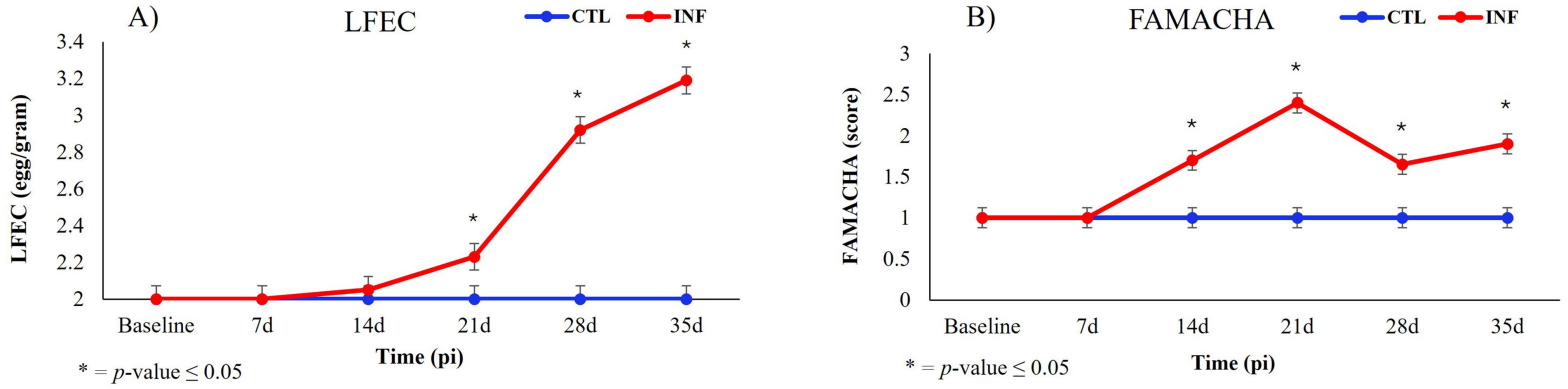
Log transformed fecal egg count (LFEC, **A**) and FAMACHA score **(B)** in *H. contortus* infected (INF) and control (CTL) Florida Cracker ewes over time [Baseline, 7-days (7d), 14-days (14d), 21-days (21d), 28-days (28d), and 35-days (35d) post-infection (pi)].

The results for LFEC, FAMACHA score, and hematological parameters are presented in Table 1. In the INF group, untransformed FECs were significantly elevated (628 ± 216 eggs/g) compared to the CTL group [0 eggs/g (*p* ≤ 0.05)]. Significant differences between INF and CTL groups were also observed for LFEC values (*p* ≤ 0.05) at 21-, 28- and 35-days pi between INF and CTL groups (Figure 2A). A significant difference (*p* ≤ 0.05) in FAMACHA score was observed between the INF (1.61 ± 0.049) and CTL (1.00 ± 0.049) groups (Table 1). The INF group consistently exhibited higher FAMACHA scores 14-, 21-, 28- and 35-days pi when compared to the CTL group (Figure 2B).

**Table 1:** Effects of *Haemonchus contortus* infection on fecal egg count (FEC), FAMACHA score, white blood cell count (WBC, 10^9^/L), lymphocyte count (LYM, 10^9^/L), monocyte count (MON, 10^9^/L), neutrophil count (NEU, 10^9^/L), lymphocyte percentage (LYM%, %), neutrophil percentage (NEU%, %), red blood cell count (RBC, 10^12^/L), hemoglobin level (HGB, g/dL), hematocrit (HCT, %), and mean corpuscular volume (MCV, fL) in infected (INF) and control (CTL) periparturient Florida Cracker ewes.

Item	Ewe group			*p*-value		
	CTL	INF	SEM	Trt	Time	Trt × Time
LFEC (eggs/gram)	2.0	2.4	0.05	<0.01	<0.01	<0.01
FAMACHA (score)	1.00	1.61	0.05	<0.01	<0.01	<0.01
WBC (10^9^/L)	6.7	7.3	0.57	0.50	<0.01	0.02
LYM (10^9^/L)	5.57	5.88	0.42	0.64	<0.01	0.02
MON (10^9^/L)	0.03	0.04	<0.01	0.61	<0.01	<0.01
NEU (10^9^/L)	0.93	1.35	0.15	0.08	<0.01	<0.01
LYM (%)	84.4	82	1.39	0.24	<0.01	<0.01
NEU (%)	13.1	18.2	1.19	<0.01	<0.01	<0.01
RBC (10^12^/L)	12.2	11.5	0.26	0.1	0.02	0.1
HGB (g/Dl)	13.2	12.9	0.39	0.51	0.06	0.97
HCT (%)	38.4	37.5	0.78	0.46	0.05	0.58
MCV (fl)	31.8	32.6	0.58	0.31	<0.01	0.01

SEM, standard error of the mean.

**Figure 3:**
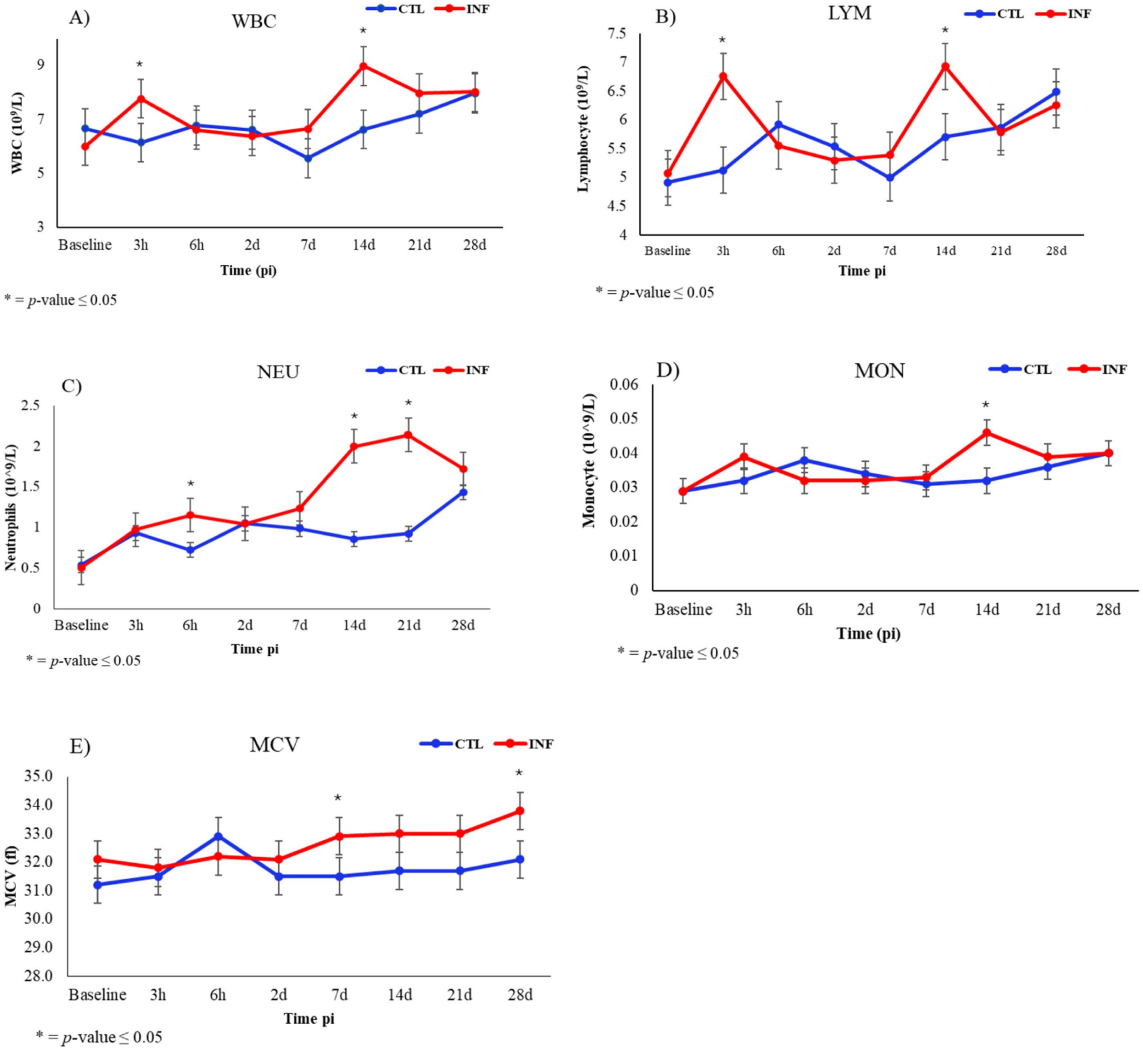
White blood cell count (WBC, **A**), lymphocyte count (LYM, **B**), neutrophil count (NEU, **C**), monocyte count (MON, **D**), and mean corpuscular volume (MCV, **E**) in *H. contortus* infected (INF) and control (CTL) Florida Cracker ewes overtime (baseline, 3-h (3 h), 6-h (6 h), 7-days (7d), 14-days (14d), 21-days (21d), and 28-days (28d) post-infection (pi)).

For hematological data, significant differences were observed among experimental groups, specifically for white blood cell count (WBC), neutrophil count (NEU), monocyte count (MON), lymphocyte count (LYM), and mean corpuscular volume (MCV) (Table 1). For both WBC and LYM, significant differences (*p* ≤ 0.05) between INF and CLT groups were found at 3-h and at 14-days pi (Figures 3A,B).

For NEU, significant variation among groups was observed at 6-h, and at 14- and 21-days pi (Figure 3C). For MON, only 14-days pi showed significant differences (*p* ≤ 0.05) between INF and CTL ewes (Figure 3D). The MCV differed significantly (*p* ≤ 0.05) only at 7- and 28-days pi between INF and CTL ewes (Figure 3E). No significant differences were observed in RBC, HGB and HCT among experimental groups (Table 1).

### 3.2 Colostrum composition

No significant differences were observed for fat, protein, lactose, total solids, solids non-fat between INF and CTL groups. Results for chemical composition are presented in Table 2.

**Table 2:** Colostrum composition.

Variables	Group	*N*	Mean	SEM	*p*-value
Fat	Infected	10	10.3	1.22	0.43
	Control	10	11.7		
Protein	Infected	10	9.48	0.5	0.66
	Control	10	9.17		
Lactose	Infected	10	2.37	0.33	0.75
	Control	10	2.52		
Total solids	Infected	10	20.4	1.39	0.46
	Control	10	21.8		
Solids non-fat	Infected	10	12.4	0.42	0.96
	Control	10	12.4		

Total fat, protein, lactose, solids, solids, and non-fat content in colostrum samples from infected (INF) and control (CTL) Florida Cracker ewes.

*N*, number of animals per group; SEM, Standard error of the mean.

### 3.3 Colostrum metabolomics

**Figure 4:**
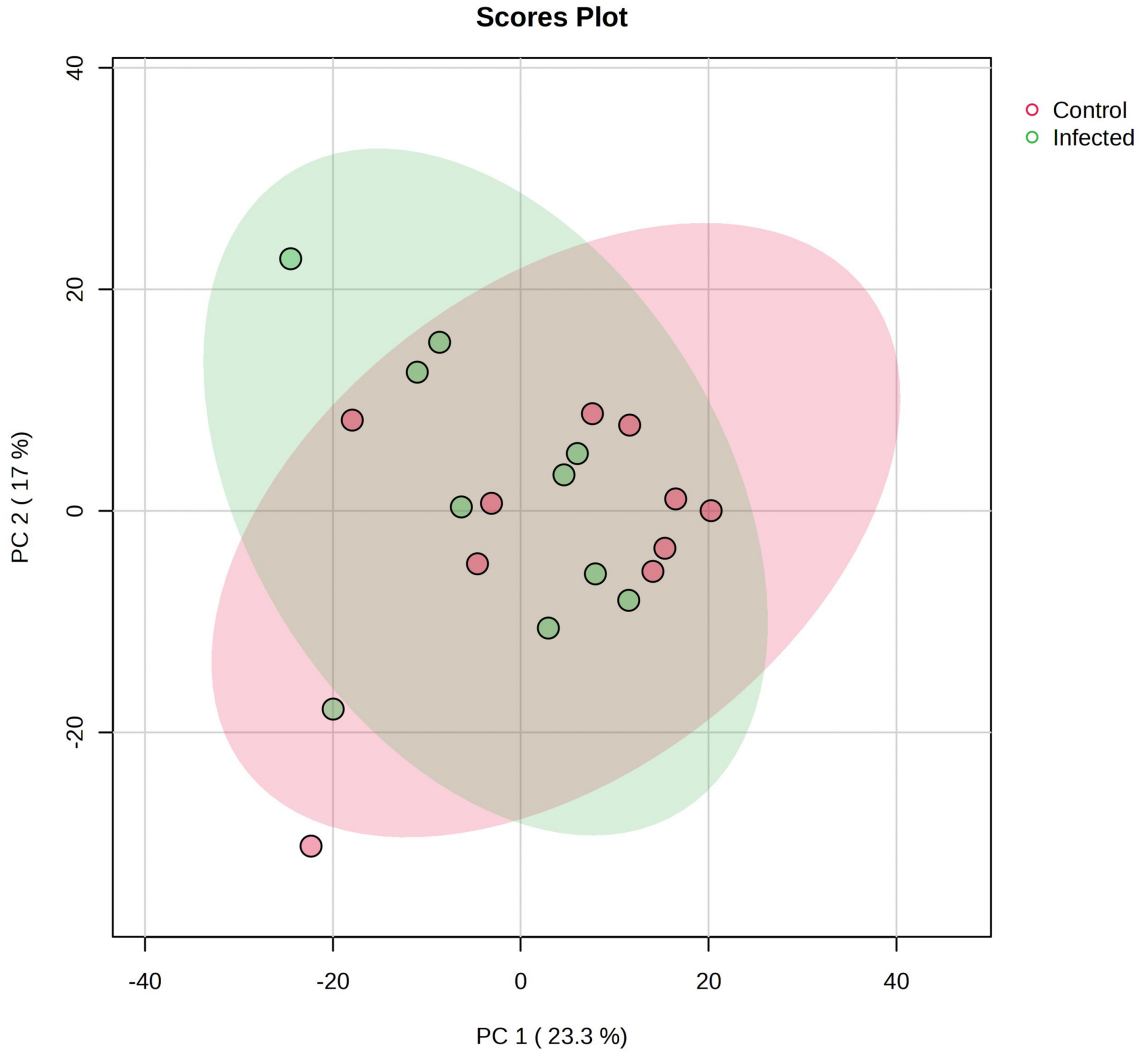
The PCA plot for colostrum metabolic data of infected (INF) and control (CTL) Florida Cracker ewes. Green represents the metabolites in the INF group. Red represents the metabolites in the CTL group.

**Figure 5:**
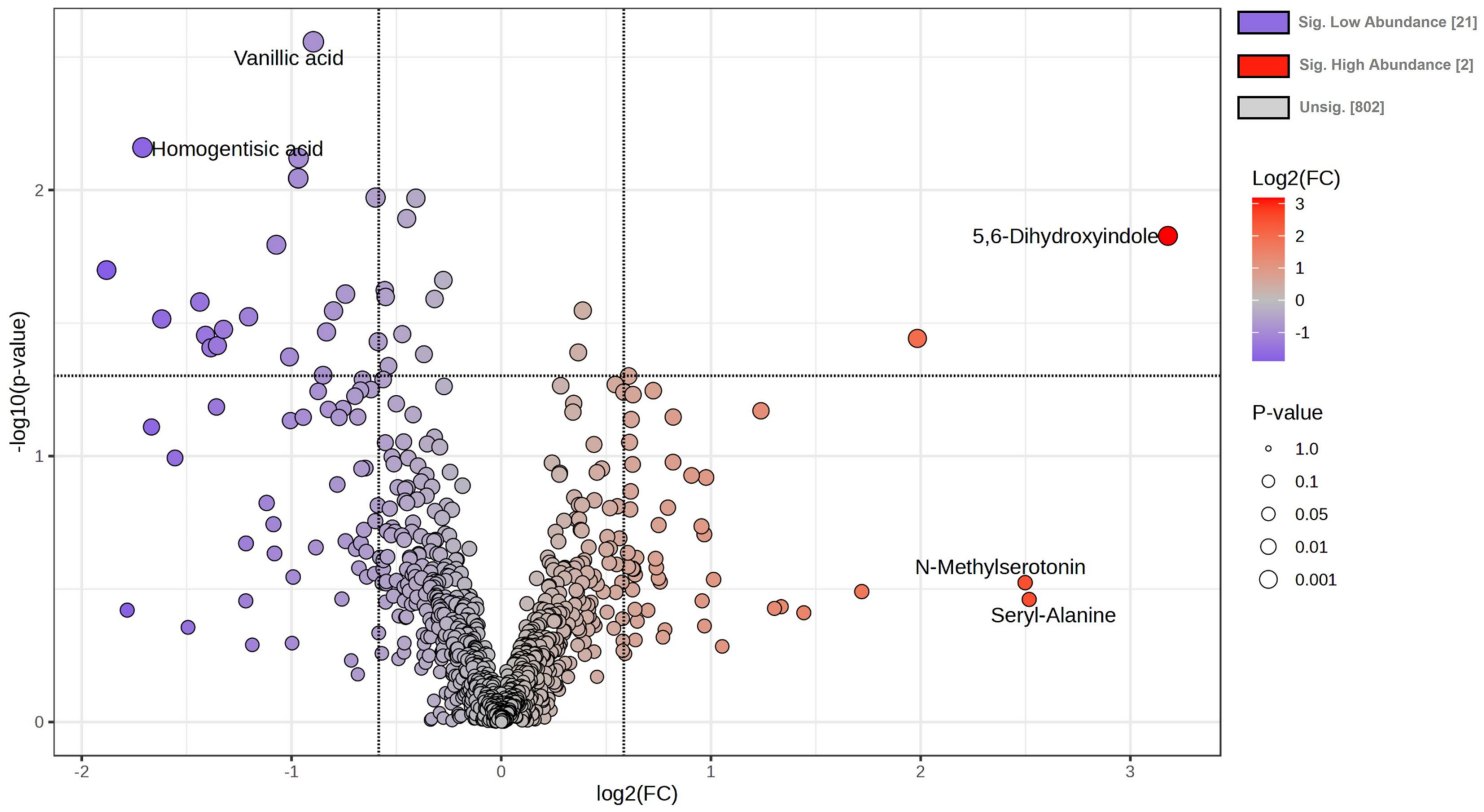
Volcano plot for differentially abundant metabolites between colostrum samples of infected (INF) and control (CTL) Florida Cracker ewes (*p* ≤ 0.05, FC ≥ 1.5 or ≤ 0.67), unequal variance.

**Figure 6:**
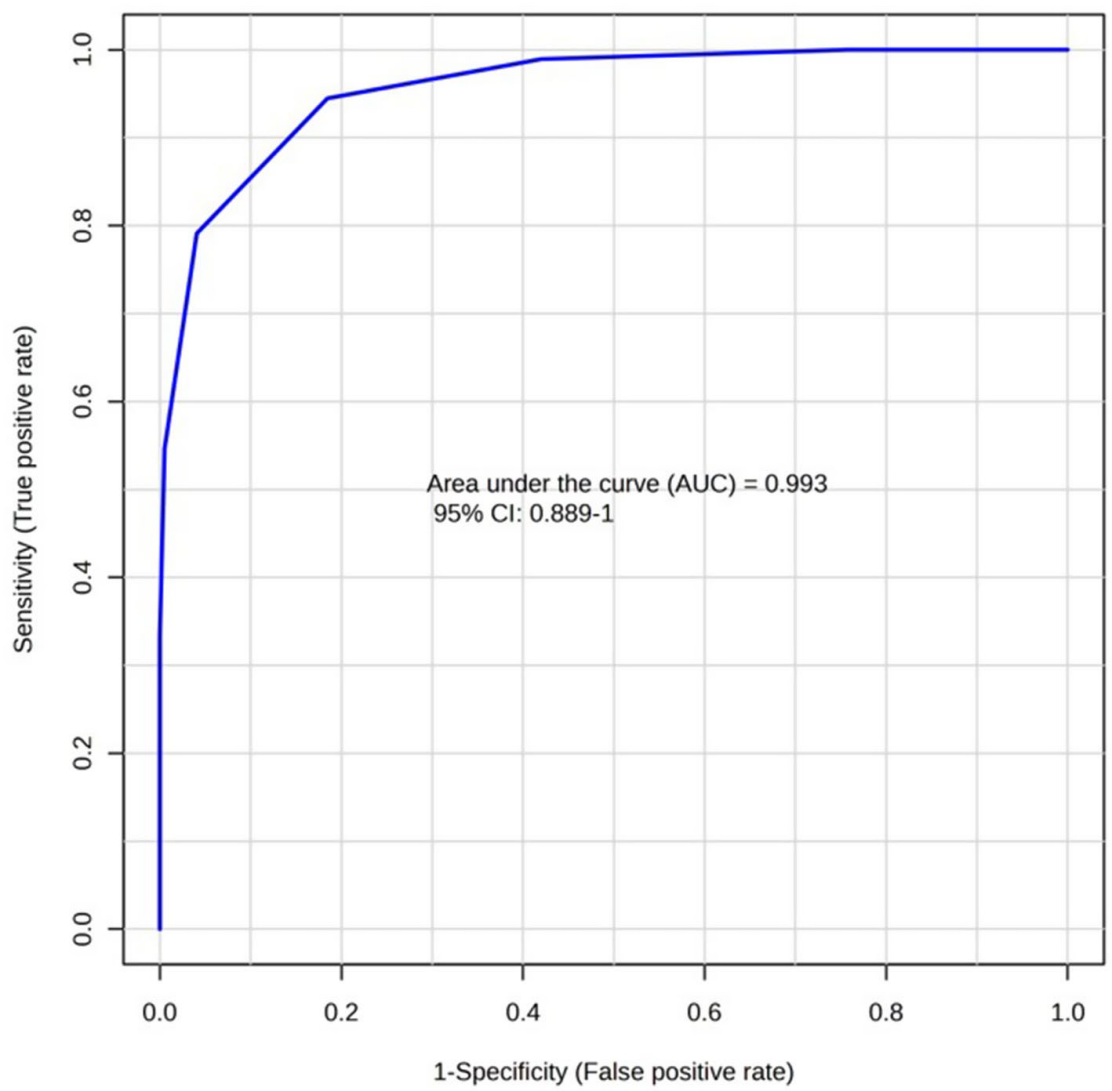
AUC curve plot showing the combined biomarkers effect of 3-sulfocatechol, pyrocatechol sulfate, guaiacol, 5,6,7,8-tetrahydromonapterin, glutamyl-tyrosine, and isomer 2 of equol between the colostrum samples of infected (INF) and control (CTL) Florida Cracker ewes with *H. contortus*.

**Figure 7:**
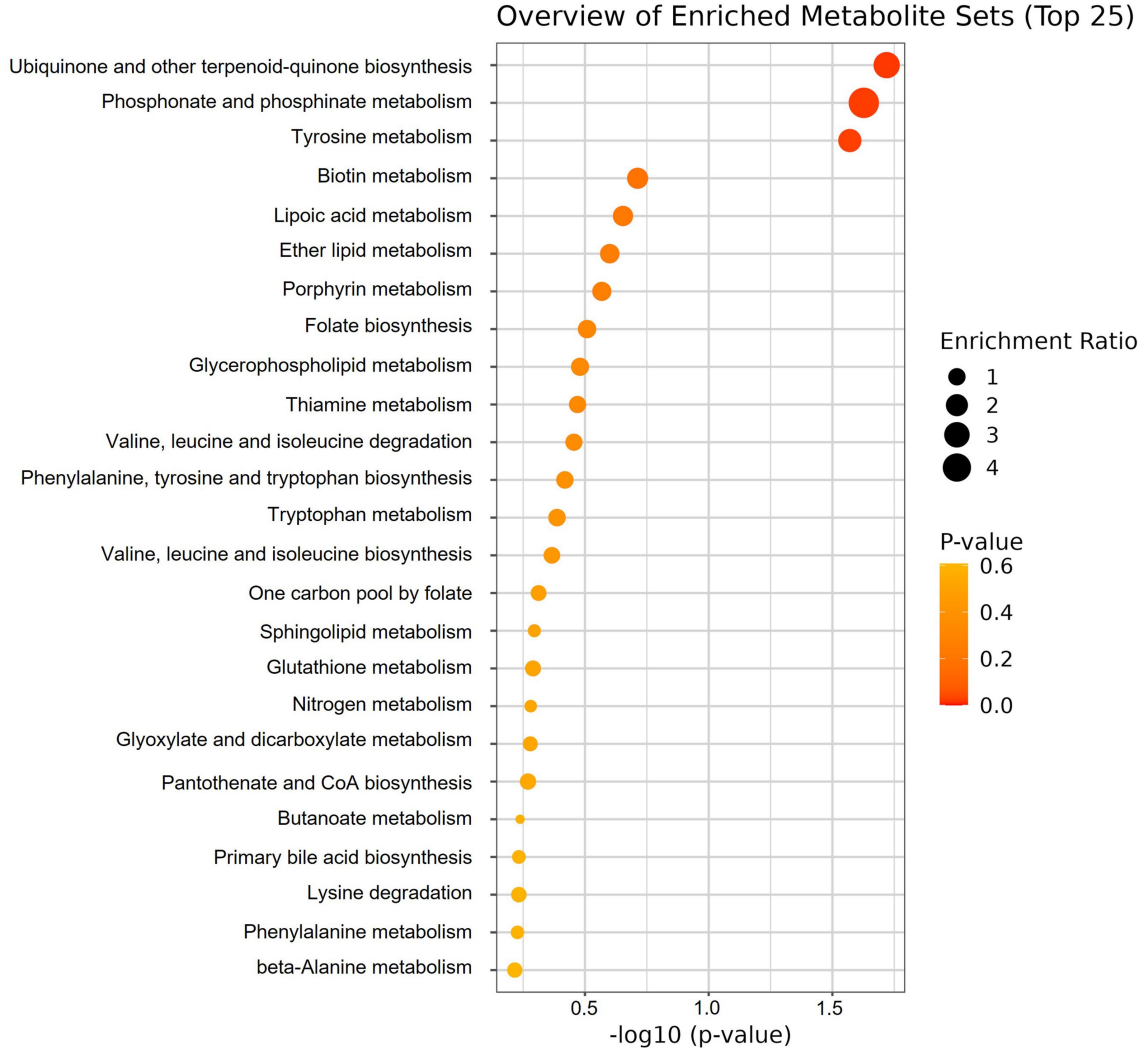
Pathway enrichment analysis for differentially abundant metabolites between colostrum samples of infected (INF) and control (CTL) Florida Cracker ewes (−log_10_ *p*-value >1.3 = *p* < 0.05).

A total number of 825 metabolites were identified in the colostrum samples (Supplementary material S3). The PCA plot showed no clear separation between INF and CTL groups. The first and second principal components explained 23.3 and 17% of the total variance observed between INF and CTL groups, respectively (Figure 4). The volcano plot analysis revealed 23 differentially abundant (*p* ≤ 0.05, FC ≥ 1.5 or ≤ 0.67) metabolites in the colostrum samples between INF and CTL groups (Figure 5). Colostrum concentrations of 21 metabolites, such as 3-(2,3-dihydroxyphenyl) propanoic acid, homogentistic acid, guaiacol and 5-carboxyvanillic acid, vanillic acid showed decreased abundance (*p* ≤ 0.05, FC ≤ 0.67) in the INF group ewes. Only two metabolites, 5,6-dihydroxyindole and arginyl-glycine, exhibited increased abundance between groups (*p* ≤ 0.05, FC ≥ 1.5). The results of the ROC analysis revealed 12 candidate biomarker panels with AUC > 0.80 and *p*-value ≤ 0.05 (Table 3). Cluster ROC analysis shows the combined effect of 3-sulfocatechol, Pyrocatechol sulfate, Guaiacol, 5,6,7,8-Tetrahydromonapterin, Glutamyl-Tyrosine and isomer 2 of equol is a better biomarker panel between the 2 groups colostrum sample (AUC > 99%) (Figure 6) than each of the individual biomarkers. Results of the pathway enrichment analysis revealed that ubiquinone and other terpenoid-quinone biosynthesis, phosphonate and phosphinate metabolism were the most differentially enriched pathways (*p* ≤ 0.05) (Figure 7).

**Table 3:** Differentially abundant metabolites with AUC > 0.80 and *p*-value ≤ 0.05 between colostrum samples from infected (INF) and control (CTL) Florida Cracker ewes.

S/N	Biomarker	AUC	T-test	Log_2_FC
1	Vanillic acid	0.87	0.003	0.87
2	3-Sulfocatechol	0.86	0.01	0.92
3	Pyrocatechol sulfate	0.86	0.01	0.92
4	Guaiacol	0.84	0.02	1.87
5	Homogentisic acid	0.84	0.005	1.73
6	5,6,7,8-Tetrahydromonapterin	0.83	0.02	0.53
7	3-(2,3-Dihydroxyphenyl) propanoic acid	0.82	0.02	1.35
8	CMP-2-aminoethylphosphonate	0.82	0.01	0.43
9	Glutamyl-Tyrosine	0.82	0.01	0.46
10	Isomer 1 of Valyl-Valine	0.82	0.01	0.58
11	Isomer 2 of Equol	0.82	0.01	0.97
12	3,4-Dihydroxyphenylvaleric acid	0.8	0.03	1.28

FC, fold change.

### 3.4 Colostrum Lipidomics

**Figure 8:**
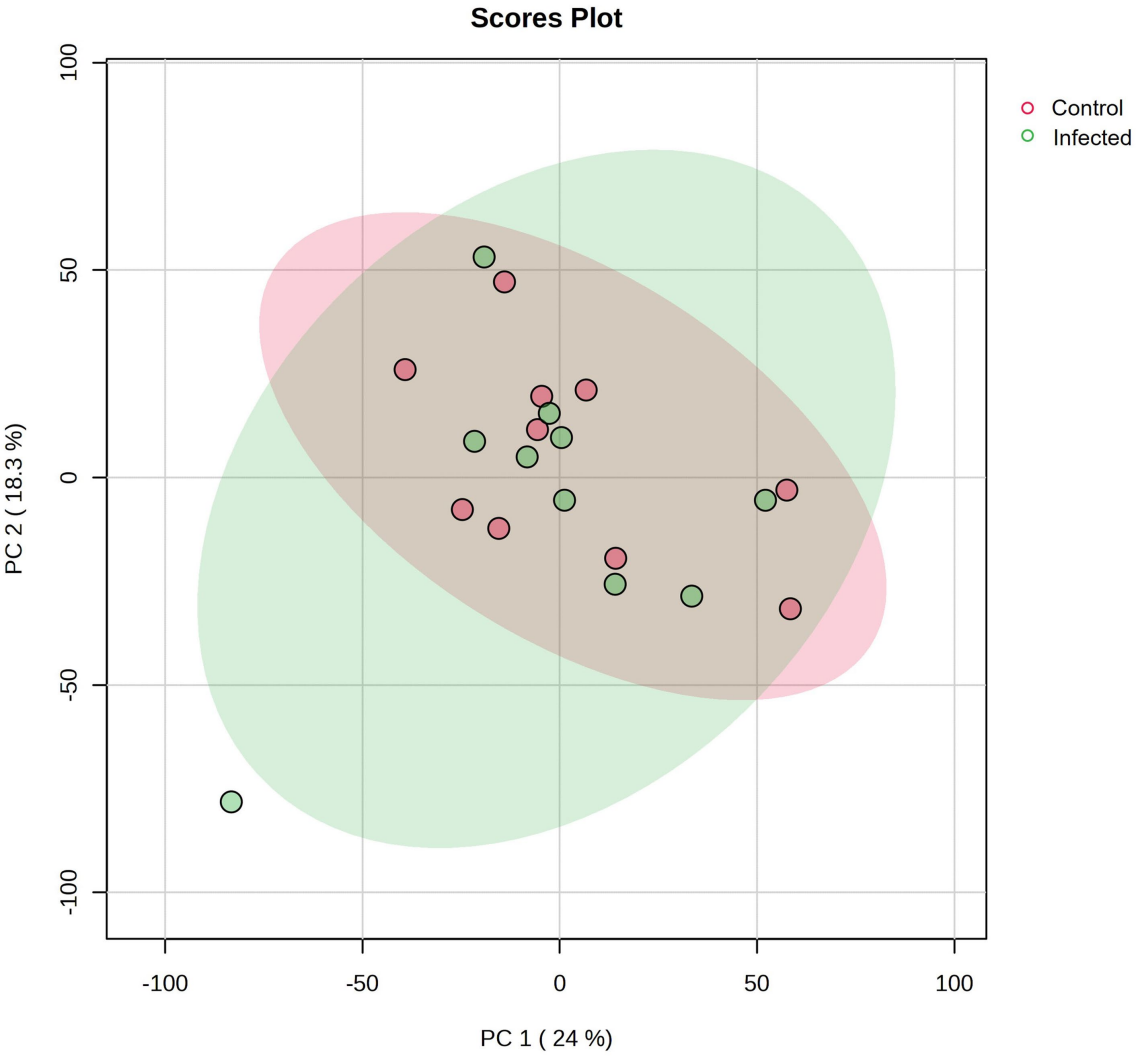
PCA plot of the colostrum lipidome of *H. contortus* infected (INF) and control (CTL) Florida Cracker ewes. Green represents the lipids in the INF group. Red represents the lipids in the CTL group.

**Figure 9:**
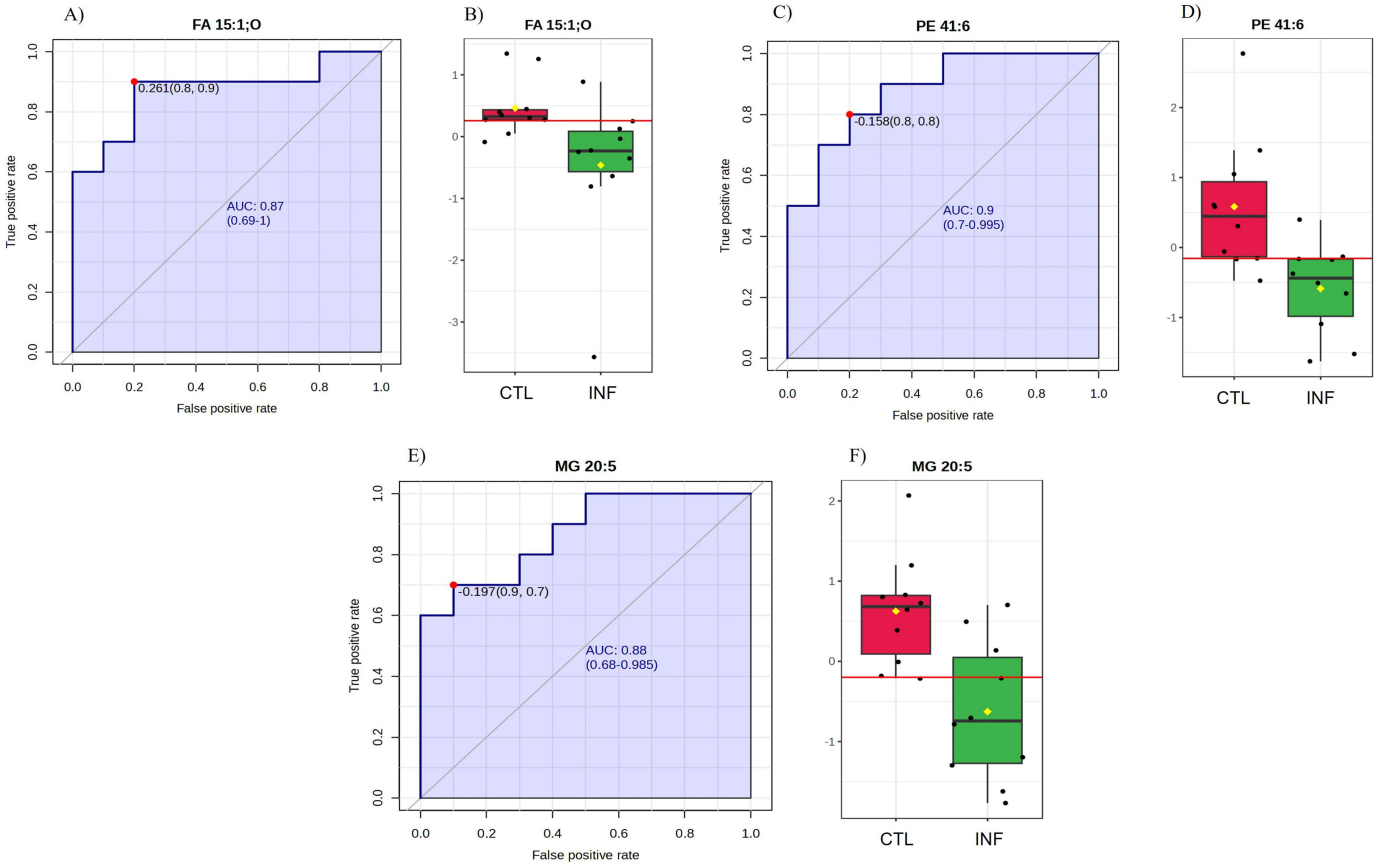
ROC curve analysis of candidate lipid biomarkers FA 15:1; O **(A)**, PE 41:6 **(C)**, and MG 20:5 **(E)** of colostrum samples from infected (INF) and control (CTL) Florida Cracker ewes. The red point label corresponds to the optimal cut-off point on the ROC curve, and the numbers in brackets represent 95% confidence intervals. Relative distributions **(B,D,F)** of the candidate lipid biomarkers of colostrum samples from infected (INF) and control (CTL) Florida Cracker ewes.

Carboxylic acid-based lipidome analysis detected and identified 4,702 lipid species in the colostrum samples (Supplementary material S4). PCA plot analyses showed no separation between the colostrum samples from INF and CTL groups (Figure 8). Biomarker analysis identified 3 lipid species (FA 15:1;O, MG 20:5, PE 41:6) as potential biomarkers among experimental groups (Figure 9).

## 4 Discussion

This study provides a comprehensive evaluation of the effect of *H. contortus* infection on hematological and parasitological parameters, as well as on the composition, metabolomic, and lipidomic profiles of colostrum in Florida Cracker ewes. To our knowledge, this is the first study to investigate the influence of *H. contortus* infection on colostrum metabolomics and lipidomics.

Florida Cracker sheep is a heritage sheep breed known for its exceptional resistance to gastrointestinal parasites (35–38). This trait is particularly valuable in combating highly pathogenic and hematophagous parasites such as *H. contortus*. FAMACHA score and FEC are common parasitological parameters evaluated during infections with *H. contortus* in sheep (39–41) and have been proposed as phenotypic markers for selection of parasite resistant individuals in Florida Cracker sheep (38). In our study, infected ewes showed significant increases in LFEC, and FAMACHA scores compared to the control group, indicating successful establishment of *H. contortus* infection. However, no significant differences were observed in RBC, HGB, and HCT levels (Table 1). These hematological parameters are commonly used as indicators of anemia in sheep infected with *H. contortus* (42). The absence of significant changes in these hematological markers underscores the parasite resistant ability of Florida Cracker sheep. A noteworthy finding in this study was the significantly elevated mean corpuscular volume (MCV) observed at 7-days pi (Figure 3E). The MCV is used to assess the average size of red blood cells and plays a crucial role in the diagnosis and morphological classification of anemia (43). The increase observed at 7-days pi may reflect a regenerative response, characterized by the production of immature, larger red blood cells, occurring without a significant decline in RBC, HGB, or HCT levels. Previous studies with Pelibuey sheep, a parasite resistant sheep breed from Mexico, have observed high MCV in infected individuals (44).

Notably, infected ewes exhibited significant differences in WBC, LYM, NEU and MON compared to the control group (Table 1). Similar results for these hematological responses have been reported in other parasite resistant sheep breeds such as St. Croix (45). For WBC and LYM, the significant increases observed at 3-h, and 14-days (Figures 3A,B) could be attributed to early immune activation and adaptive mechanisms, respectively. Previous studies with Florida Cracker sheep have highlighted the role of lymphocyte immune responses, particularly those involving Th17, Treg, and Th2 pathways, in conferring resistance to *H. contortus* infection (35). For NEU, significant increases in the infected ewes were observed at 6-h, 14-days and 21-days pi (Figure 3C). Neutrophils are the most abundant leukocytes in peripheral blood and serve as first responders of the innate immunity during infections. Neutrophilia is commonly observed in infected sheep with *H. contortus* (45, 46) and the presence of neutrophils has been associated with parasite clearance (47). Another interesting observation in this study is the significant increase in MON detected at 14-days pi (Figure 3D). Monocytes circulating in peripheral blood serve as precursors to macrophages and dendritic cells (48) and elevated monocyte counts have been reported in the peripheral blood of sheep infected with *H. contortus* (46). The presence of monocytes has also been associated with inflammatory responses that may contribute to increased larval morbidity and reduced motility, potentially impairing parasite survival and development (49).

Results from the chemical composition analysis of colostrum indicated that *H. contortus* infection did not alter its composition in the experimental ewes (Table 2). Although previous studies have investigated the impact of gastrointestinal parasites on sheep milk composition (11, 13), their effect on colostrum composition remains unexplored.

Assessment of colostrum metabolites provides an intricate insight into the metabolic adaptations that occur in ewes under infection-induced stress. Since colostrum constituents are primarily sourced from circulating plasma constituents (6), it is possible that changes in the metabolite profile can serve as sensitive indicators of metabolic alterations resulting from infection. Therefore, metabolomic analysis of colostrum facilitates the identification of specific biochemical pathways affected by infection, thereby enhancing the understanding of host-pathogen interactions at the metabolic level.

Previous studies in dairy cattle have reported alterations in milk metabolites in response to mastitis and have proposed several of these compounds as potential diagnostic biomarkers for this health condition (50–52). However, most of this work has focused on conventional milk metabolites and has not examined metabolomic signatures present in colostrum. Our study is the first to investigate changes in colostrum metabolites and lipids associated with *H. contortus* infection in sheep. Metabolomic profiling identified a total of 825 metabolites, of which 23 were differentially abundant (Figure 5), with most displaying decreased levels in infected ewes. Several of these compounds, including homogentisic acid and dihydroxyindole are linked to antioxidant, antiparasitic and anti-inflammatory effects (24, 53, 54). Notably, arginyl-glycine and 5,6-dihydroxyindole were significantly more abundant in colostrum samples from infected ewes. The presence of dipeptides such as arginyl-glycine in colostrum may result of milk protein hydrolysis. In bovine milk, bioactive peptides such as arginyl-glycine have exhibited antioxidant, immunomodulatory properties and mucosal immunity protection of the gastrointestinal tract (55). The presence of arginyl-glycine in colostrum may contribute to the immune protection of lambs during infection with *H. contortus*. Further research is required to clarify the role of arginyl-glycine in the colostrum of infected ewes and to assess their influence on lamb survival and nutritional status. The metabolite 5,6-dihydroxyindole showed the highest difference between the 2 groups and it is directly involved in tyrosine metabolism. This metabolite is a downstream derivative of tyrosine and undergoes enzymatic modification in the synthesis of eumelanin (56–59). Eumelanin possesses antioxidative and free radical scavenging properties (60, 61). It also interacts with melanin in biological systems to stimulate the synthesis of immune cells (62–64). Dihydroxyindole has also shown antiparasitic, antiviral and antibiotic properties during insect immune responses (24). Additionally, dihydroxyindole oxidation was also observed to impede parasite survival in insects (65). Thus, it is possible that the presence of dihydroxyindole detected in the colostrum of *H. contortus*-infected ewes could play a role in host defense mechanisms targeting the parasite. More studies are needed to elucidate the role of dihydroxyindole in the colostrum of infected ewes with *H. contortus* and its benefits for lamb survival.

Twenty-one metabolites such as guaiacol, homogentisic acid, and homogentisic acid 2-o-sulfate were significantly less abundant in the infected group. Guaiacol is a phenolic metabolite derived from lignin, and it is known for its antioxidant activity (66) and free radical scavenging ability (67). Homogentisic acid is a metabolite that is involved in the metabolic degradation of tyrosine through the action of the enzyme homogentisate 1, 2-dioxygenase (68, 69) and it has been recognized for its antioxidant properties (53, 54). In sheep, *H. contortus* infection triggers oxidative stress and increases the reactive oxygen species due to blood loss and abomasal tissue damage (3, 70, 71). Thus, it is possible these metabolites may be consumed more rapidly due to their antioxidant activities, as they may help to counteract the oxidate stress caused by the parasite. This increased utilization may result in lower concentrations of these compounds in colostrum. Cluster biomarker analysis showed that the combined panel of 3-sulfocatechol, pyrocatechol sulfate, guaiacol, 5, 6, 7, 8-tetrahydropterin, glutamyl-tyrosine, and isomer 2 of equol provided superior discrimination between infected and control colostrum samples compared with individual metabolites. It is important to acknowledge that the concentrations of these metabolites may vary as a function of dietary composition. In the present study, both the infected and control groups were maintained on an identical diet to minimize this source of variation. Nonetheless, further investigations are needed to validate these candidate biomarkers and further confirm our findings.

**Figure 10:**
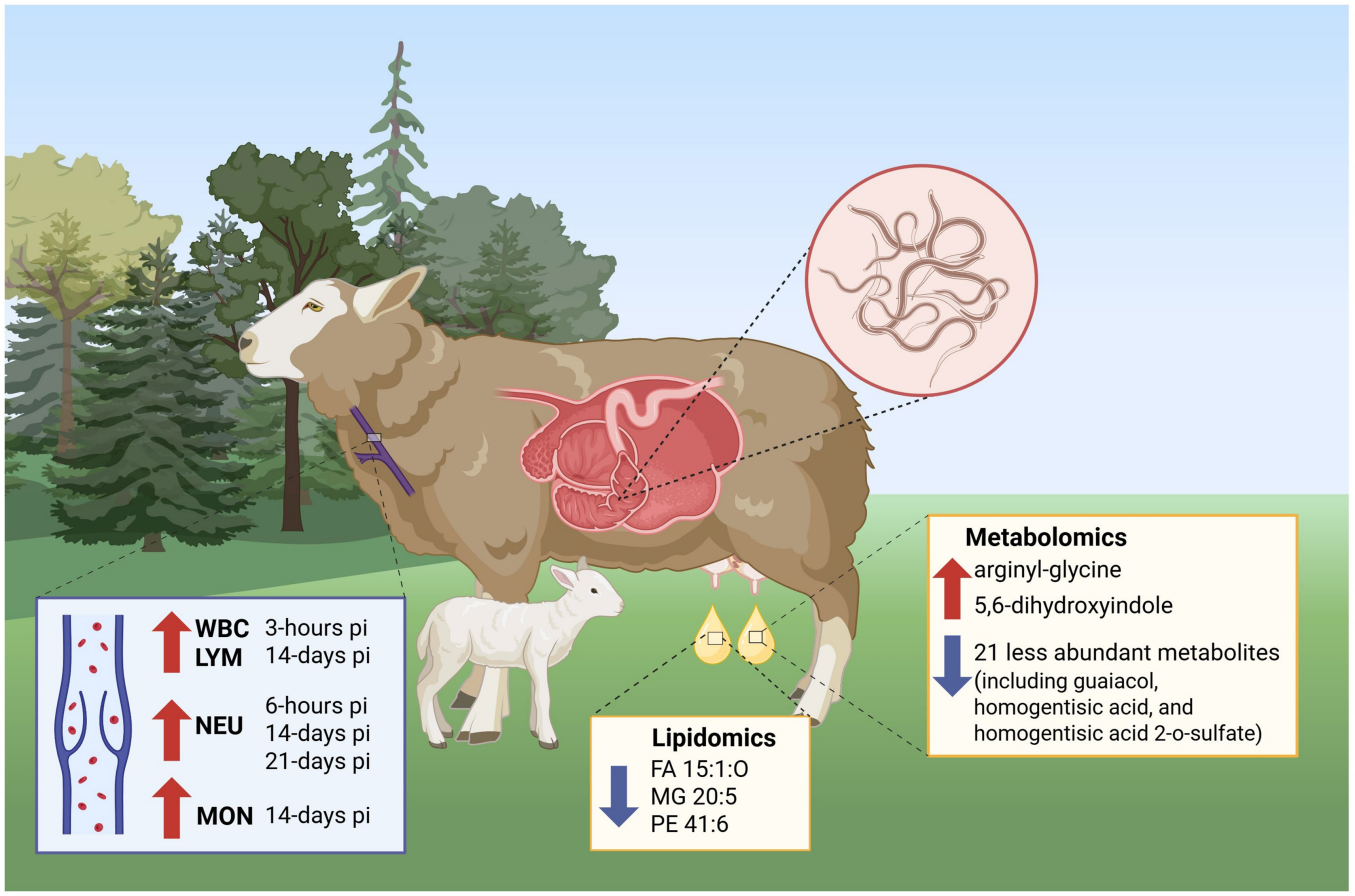
Alterations in hematological parameters, colostrum lipidome, and metabolome in infected Florida Cracker ewes with *H. contortus.* Image was created with BioRender. Estrada, Z. (2026) https://BioRender.com/ogs2uwi

Metabolic pathway analysis (Figure 7) influenced by quantified metabolites revealed that ubiquinone and other terpenoid-quinone biosynthesis, phosphonate and phosphinate metabolism, and tyrosine metabolism were the most differentially enriched pathways. The enrichment of the tyrosine metabolism pathway may be linked to its role in immune stimulation through the production of antibodies, cytokines, and the regulation of immune cells (72, 73), potentially serving as a coping mechanism to help the infected ewes activate their immune response during infection with *H. contortus*. Ubiquinone is a powerful antioxidant that plays an essential role in energy metabolism via the mitochondrial respiratory chain, aiding in the production of adenosine triphosphate. The ubiquinone and terpenoid-quinone pathways are linked to oxidative stress regulation, as they are involved in the generation of redox-active compounds, including ubiquinone and other related quinones (74). Phosphonates are organophosphorus compounds that have demonstrated antibacterial and anti-inflammatory properties (75). The significant enrichment of these pathways in colostrum may reflect the ewe’s physiological response to infection, which alters blood constituents and subsequently affects the composition of colostrum metabolites (Figure 10).

Previous studies have demonstrated that *H. contortus* infection induces oxidative stress in the host (3, 70, 71), which can disrupt ruminal lipid metabolism (76). These metabolic alterations may, in turn, influence the lipid profile involved in colostrum synthesis. Results from biomarker lipid analysis identified three lipid species (FA 15:1:O, MG 20:5, PE 41:6) as potential biomarkers between the infected and control groups. Pentadecanoic acids such as FA 15:1:O, have been suggested as a potential fatty acid possessing anti-inflammatory, antifibrotic, red blood cell-stabilizing activities to attenuate anemia *in vivo* (77, 78). Research evidence has also highlighted the importance of this fatty acid for liver health (77–79). Additionally, alterations of the liver lipids have been reported in lambs infected with *H. contortus* (76). The MG 20:5 is a monoacylglycerol which is an intermediate in the biosynthesis of phospholipids and is vital for many cellular processes (80). The PE 41:6 is a type of phosphatidylethanolamines, which are major phospholipids (15%–25%) in mammalian cells (81). Phosphatidylethanolamines play many functional roles in several cellular processes (82, 83). This includes protein biogenesis (82, 83), oxidative phosphorylation (83–85) and maintains a critical balance with other phospholipids to ensure liver well-being (86). These membrane phospholipids have a significant impact on mitochondria structure and function (87), and their supply in colostrum has been suggested as supportive of energy biogenesis in growing tissues of calves (5).

## 5 Conclusion

In conclusion, our study demonstrates that infection with *H. contortus* influences the parasitological and hematological responses of periparturient ewes and alters the colostrum metabolome and lipidome profile. Although no differences in chemical composition of colostrum were observed, significant metabolites and lipids were identified. The observed parasitological and hematological responses, along with the elevated levels of antioxidative metabolites, suggest an adaptive physiological response in infected ewes aimed at mitigating parasite burden. This response appears to influence the metabolite composition of their colostrum. Future studies are needed to understand the key role of the identified metabolites and lipids altered by *H. contortus* infection and their impact on lamb performance.

## Data Availability

The datasets presented in this study can be found in online repositories. The names of the repository/repositories and accession number(s) can be found in the article/Supplementary material.

## Footnotes

1
https://fiehnlab.ucdavis.edu/projects/LipidBlast

2
https://mona.fiehnlab.ucdavis.edu

3
http://www.lipidmaps.org
